# Fluid Mechanical Effects of Fetal Aortic Valvuloplasty for Cases of Critical Aortic Stenosis with Evolving Hypoplastic Left Heart Syndrome

**DOI:** 10.1007/s10439-023-03152-x

**Published:** 2023-02-13

**Authors:** Hong Shen Wong, Binghuan Li, Andreas Tulzer, Gerald Tulzer, Choon Hwai Yap

**Affiliations:** 1 Department of Bioengineering, Imperial College London, Exhibition Road, London, SW7 2AZ UK; 2 Department of Pediatric Cardiology, Children’s Heart Center Linz, Kepler University Hospital, Krankenhausstrasse 26-30, 4020 Linz, Austria; 3 Medical Faculty, Johannes Kepler University Linz, Altenberger Strasse 69, 4040 Linz, Austria

**Keywords:** Fluid mechanics, Fetal cardiology, Fetal aortic stenosis and evolving hypoplastic left heart syndrome, Fetal cardiac intervention

## Abstract

Fetuses with critical aortic stenosis (FAS) are at high risk of progression to HLHS by the time of birth (and are thus termed “evolving HLHS”). An *in-utero* catheter-based intervention, fetal aortic valvuloplasty (FAV), has shown promise as an intervention strategy to circumvent the progression, but its impact on the heart’s biomechanics is not well understood. We performed patient-specific computational fluid dynamic (CFD) simulations based on 4D fetal echocardiography to assess the changes in the fluid mechanical environment in the FAS left ventricle (LV) directly before and 2 days after FAV. Echocardiograms of five FAS cases with technically successful FAV were retrospectively analysed. FAS compromised LV stroke volume and ejection fraction, but FAV rescued it significantly. Calculations to match simulations to clinical measurements showed that FAV approximately doubled aortic valve orifice area, but it remained much smaller than in healthy hearts. Diseased LVs had mildly stenotic mitral valves, which generated fast and narrow diastolic mitral inflow jet and vortex rings that remained unresolved directly after FAV. FAV further caused aortic valve damage and high-velocity regurgitation. The high-velocity aortic regurgitation jet and vortex ring caused a chaotic flow field upon impinging the apex, which drastically exacerbated the already high energy losses and poor flow energy efficiency of FAS LVs. Two days after the procedure, FAV did not alter wall shear stress (WSS) spatial patterns of diseased LV but elevated WSS magnitudes, and the poor blood turnover in pre-FAV LVs did not significantly improve directly after FAV. FAV improved FAS LV’s flow function, but it also led to highly chaotic flow patterns and excessively high energy losses due to the introduction of aortic regurgitation directly after the intervention. Further studies analysing the effects several weeks after FAV are needed to understand the effects of such biomechanics on morphological development.

**Supplementary Information:**

The online version contains supplementary material available at 10.1007/s10439-023-03152-x.

## Introduction

Hypoplastic Left Heart Syndrome (HLHS) is a severe congenital heart disease characterised by critically underdeveloped left heart structures,^27^ incapacitating cardiac functions such that the heart is unable to efficiently perfuse the systematic circulation. HLHS is likely heterogeneous in aetiology. In a subset of HLHS patients, the fetal heart is normally grown at mid-gestation, but fetal aortic stenosis abnormality occurs at mid-gestation, together with specific abnormalities such as left ventricular dysfunction, retrograde transverse aortic arch and foramen ovale flow, and monophasic mitral valve (MV) inflow, and this causes poor growth for the remainder of gestation, leading to HLHS at birth.^3,14^

In such cases, several previous natural history studies showed that only 19–38% of fetal aortic cases achieved a biventricular (BV) postnatal outcome, and the rest had a univentricular (UV) outcome.^6,10,19^ For this reason, they are known as fetal critical aortic stenosis and evolving HLHS (FAS) by previous investigators. “Evolving HLHS” referred to their high risk of progressing to HLHS at birth, but at the time of the fetal evaluation, the left ventricle (LV) is not hypoplastic.

An *in-utero* catheter-based fetal heart intervention, fetal aortic valvuloplasty (FAV) can be performed on FAS to relieve the critical aortic stenosis, and this has demonstrated effectiveness in preventing the progression to HLHS morphology by birth.^16,24^ Multiple recent retrospective studies of FAV procedures reported that a technical success rate of 74–92% was achieved, which depended on clinician experience, and 50–71% of all liveborn fetuses after successful FAV had a BV circulation postnatal outcome.^3,10,16,24,28^

The biomechanical environment of the fetal heart is drastically altered in FAS disease and after FAV intervention. During disease, there is a drastic elevation of ventricular pressure, diminished myocardial strains and stroke volume (SV), and mitral regurgitation (MR) that elevates left atrial (LA) pressure.^8,16,17^ With a successful intervention, the LV depressurizes, LV exhibits higher strains and SV, diastolic function improves, and there is a transition from retrograde to antegrade flow in the transverse aortic arch.^4,8,26,34^ These biomechanical factors are likely to influence morphological development and birth outcomes. We have previously characterized the fluid mechanics of diseased FAS LV and found altered vorticity dynamics, wall shear stress (WSS) spatial patterns, excessive flow energy losses, and poor turnover of blood.^35^ However, a detailed investigation of the biomechanical impact of FAV has not been conducted, and our current understanding of its impact is limited to echocardiographic measurements with limited resolution.

Therefore, in this study, computational fluid dynamic (CFD) simulations of FAS LVs to characterize their biomechanical impact was conducted. Cases of technically successful FAV were studied retrospectively, and patient-specific comparisons of pre- and post-FAV conditions were performed.

## Material and Methods

### Patient Data Collection

4D ultrasound data of five FAS fetal subjects before and after the FAV procedure were prospectively collected at the Kepler University Hospital, Austria. The gestational ages ranged from 22 to 32 weeks. The pre-FAV scans were conducted the same day as the procedure, while the post-FAV scans were acquired 2 days after the intervention. The study was approved by IRB protocol number 1009/2017, with consent from the patient’s parent. The characteristics of the five FAS patients are listed in Table 1.

**Table 1:** Characteristics of aortic stenosis with evolving hypoplastic left heart syndrome (FAS) subjects before and after aortic valvuloplasty, postnatal outcomes of the same subjects and postnatal procedures undertaken.

Case	GA at scan, (week + day)	Bradycardia	LV thrombus	Pericardial effusion	Hydrops	Postnatal circulation	Outcome	Postnatal procedures
FAS 1	22 + 4	N	Y	N	N	UV	Infant death	NW
FAS 2	24 + 6	Y	N	N	N	BV	Alive	AV Dil
FAS 3	29 + 1	N	N	N	Y	BV	Alive	RK
FAS 4	29	N	N	N	Y	BV	Alive	RK
FAS 5	30 + 1	N/A	N/A	N/A	N	BV	Alive	–

GA – gestational age; LV – left ventricle; BV – biventricular; UV – univentricular; RK – Ross-Konno procedure; AV Dil. – Aortic valve dilation; NW – Norwood procedure; Y – yes; N – no; NA – not available

### Image Acquisition and Processing

Ultrasound acquisition, image processing and geometry reconstruction methods were established and outlined in the previous studies.^12,33^ Concisely, the ultrasound systems Vivid-7^®^, Vivid E9^®^, or Vivid E95^®^ (GE Healthcare, Chicago, IL, USA) were adopted to acquire 4D B-mode images during the pre-and post-FAV checks. Images were taken *via* the Spatio-Temporal Image Correlation (STIC) mode with a sweeping period of 10–15 s and a capture rate of 70–90 frames per second, giving 37–40 volumes per cardiac cycle. The 4D scans were further extracted as a stack of 2D images with a 0.5 mm step size *via* 4D View software (GE Healthcare, Chicago, IL, USA). The endocardium and cavity of the ventricles and left atrium (LA) were segmented with a custom-written lazy-snapping algorithm.^13^ The geometries were reconstructed using Vascular Modeling Toolkit software and smoothed using Geomagic Studio (Geomagic Inc., Morrisville, NC, USA).

The motion of the cardiac chambers was estimated *via* a validated cardiac motion estimation algorithm.^31^ Briefly, the algorithm fits a global motion model consisting of spatial b-splines of temporal Fourier to displacement fields obtained from pair-wise image registration of various time points, which enforces cyclic motion and spatial consistency regularizations, and the algorithm has been validated on ultrasound data and Magnetic Resonance Imaging truths.

### Computational Fluid Dynamics Simulations

CFD simulations of the LVs were conducted in ANSYS FLUENT 2019 R2 (ANSYS Inc., Canonsburg, PA, USA); the detailed methods can be found in our previous studies.^12,33^ In brief, the LV cavity geometry was meshed with 1–1.5 million tetrahedral elements with ANSYS mesh editor to ensure mesh convergence, as determined *via* WSS results.^32^ Blood density was assumed to be fixed at 1060 kg/m^3^, and dynamic blood viscosity was modelled using the Carreau-Yasuda model.^12^ Subsequently, the three-dimensional Navier–Stokes equation was solved *via* the finite volume built-in solver, with a setting of 400 timesteps per cardiac cycle for at least three cycles to minimise the effects of the stagnant initial condition. A user-defined function was used to prescribe the geometrical wall motion, as obtained from our motion-tracking algorithm. A convergence criterion was applied to ensure all scaled residuals are less than 10^–4^.

The opening and closing of valves were idealized as an instantaneously switching between an opened boundary with uniform pressure obtained from the LPM and a closed wall boundary condition. During diastole, the aortic valve (AV) orifice was closed, and the MV orifice had a prescribed pressure calculated from a lumped parameter model (LPM). Conversely, valve conditions were reversed during systole. AV and MV insufficiencies were modelled as an open orifice zone, centred on the MV or AV orifices. AV insufficiency occurred in all the examined cases after AV balloon dilation. There was not sufficient resolution to determine valve morphology, orifice shape and sizes. As such, orifice sizes were determined *via* iterative CFD simulation, where we adjusted the orifice sizes of the inlet, outlet and regurgitation orifices and re-performed the simulation until a satisfactory match of the CFD simulated valve velocities and the patient-specific Doppler velocities, including aortic outflow velocity, peak E- and A-wave inflow velocities, peak MR and aortic regurgitation (AR) velocities, was achieved.

### Lumped Parameter Model of Human Fetal Circulation

**Figure 1:**
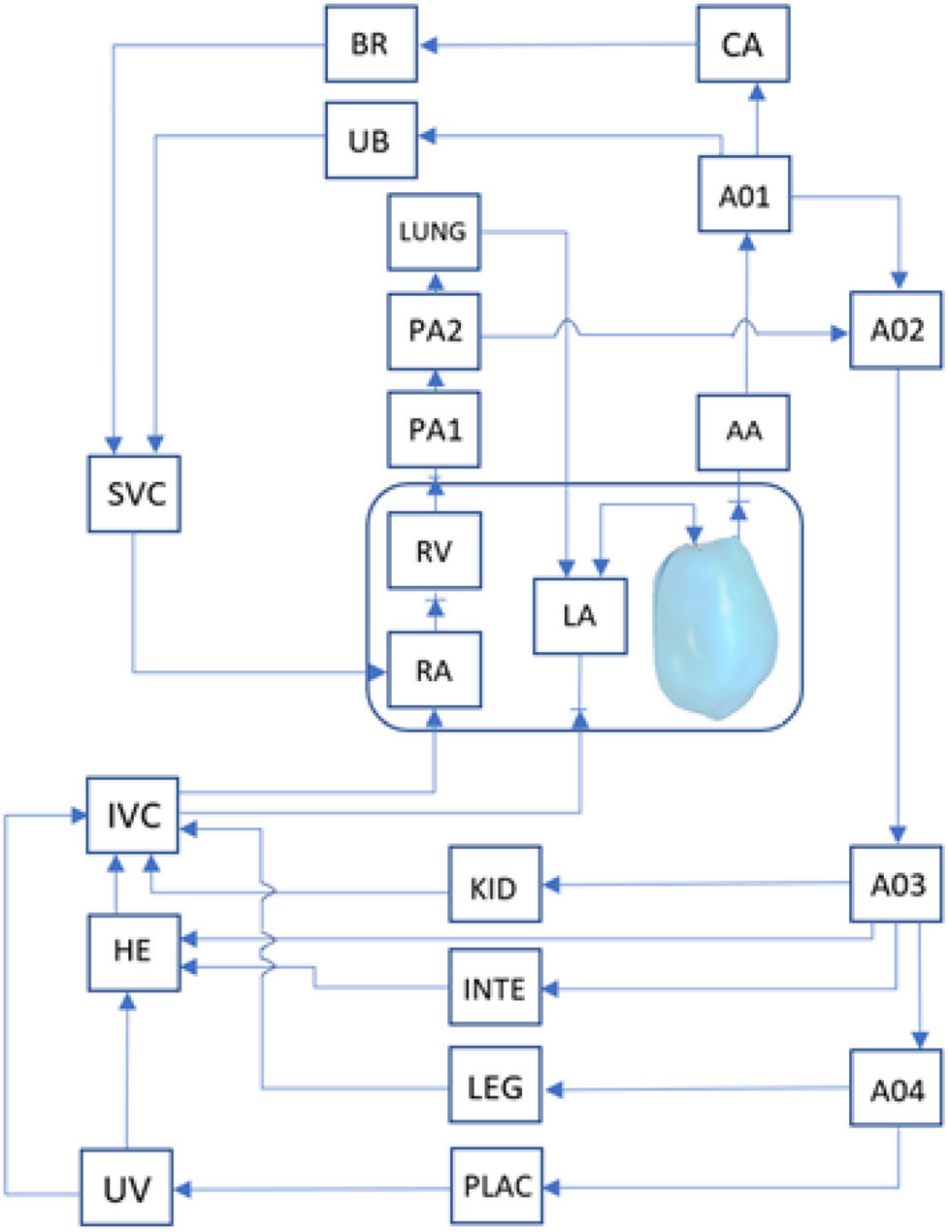
Scheme of the human fetal circulation lumped parameter model coupled to the CFD model of the left ventricle, adapted from Pennati *et al.*^21^ AA: Ascending aorta, AO1: aortic arch, AO2: thoracic descending aorta, AO3: abdominal descending aorta, AO4: femoral descending aorta, BR: brain, CA: cerebral arteries, HE: liver, INTE: intestinal circulation, IVC: inferior vena cava, KID: kidney, LA: left atrium, LEG: lower limbs, LUNG: lungs, PA1: main pulmonary artery, PA2: pulmonary arteries, PLAC: placenta, RA: right atrium, RV: right ventricle, SVC: superior vena cava, UB: upper body, UV: umbilical vein.

The implemented LPM is illustrated in Fig. 1 and was described in our previous study, where all model parameters are given.^35^ The model was originally proposed by Pennati *et al.*,^21,22^ but was recalibrated to match more recent human fetal clinical measurements, including fetal abdominal aortic pulse pressures across gestational ages, and systolic and diastolic LV pressure across gestational ages.^9,30^

The model could be scaled to different gestational ages from 22 to 38 weeks of gestation, using age-dependent factors for resistances, compliances, inertances and valvular dissipative parameters. The methodology for allometric scaling is described as,

1 $${R}_{\rm GA}={R}_{38}{\left(\frac{{W}_{\rm GA}}{{W}_{38}}\right)}^{-1}$$

2 $${C}_{\rm GA}={C}_{38}{\left(\frac{{W}_{\rm GA}}{{W}_{38}}\right)}^{1.33}$$

3 $${L}_{\rm GA}={L}_{38}{\left(\frac{{W}_{\rm GA}}{{W}_{38}}\right)}^{-0.33}$$

4 $${K}_{\rm GA}={K}_{38}{\left(\frac{{W}_{\rm GA}}{{W}_{38}}\right)}^{-1.33}$$

5 $${\mathrm{log}}_{10}{W}_{\rm GA}=0.2508+0.1458 \text{GA} - 0.0016 \text{GA}^{2}$$

where GA is the gestational age, *W* is the fetal weight, *R*, *C*, *L* and *K* were the resistance, compliance, inertances, and valvular dissipative coefficient of an element in the lumped parameter model at the 38th week of gestation or *i*th week of gestation, respectively.

The enlarged LA in disease cases indicates elevated pressures, as such an additional pressure source was modelled at the LA in the lumped parameter model as the excess volume multiplied by the normal LA compliance. Two-way coupling between the CFD model and the LPM was achieved by coding the LPM as a CFD user-defined function. Flow rates at the valve orifices from CFD were used by the LPM to evaluate pressures at the ascending aorta and the LA. These pressures were in turn used as a surface-averaged pressure boundary condition at the valve orifices in the CFD model when valves were open.

### Quantification of Energy Dynamics and Wall Shear Stress

Analysis was performed subsequently following the CFD simulations. The normalized energy loss across time, $\epsilon,$ was calculated as

6 $$\epsilon ={\int }_{T} \frac{Wd\left(t\right)+ \text{KE}_{\rm bulk}+\text{KE}_{\rm MV}+\text{KE}_{\rm AV}+\text{KE}_{\rm MR} (+\text{KE}_{\rm AR})}{\text{SV}}\;dt$$

where *t* is time, *T* is the cardiac cycle duration and SV is the stroke volume. In the expression, *Wd*(*t*) was the systolic work done by the ventricle wall, with the following expression,

7 $$Wd\left(t\right)={\int }_{{\rm CS}\left(t\right)} P \left(\overrightarrow{v} \cdot \widehat{n}\right) \;dA$$

where *P* is the pressure, $\left(\overrightarrow{v}\cdot \widehat{n}\right)$ is the dot product of the velocity vector with the surface normal, CS is the control surface (endocardium) and *A* is the surface area of the control surface. KE_bulk_ is the change in kinetic energy of the fluid in the ventricle,

8 $${\mathrm{KE}}_{\mathrm{bulk}}= \frac{d\left({\int }_{{\rm CV}\left(t\right)}^{ }\frac{1}{2}\rho |\overrightarrow{v}{\left(t\right)|}^{2} dV\right)}{dt}$$

with CV representing the control volume, $\rho$ is the blood density, $|\overrightarrow{v}{\left(t\right)|}$ is the absolute value of the velocity vector and $V$ is the volume of the control volume. Finally, ${\mathrm{KE}}_{\mathrm{MV}}$, ${\mathrm{KE}}_{\mathrm{AV}}$ and ${\mathrm{KE}}_{\mathrm{MR}}$ and ${\mathrm{KE}}_{\mathrm{AR}}$ are the kinetic energy of the flux through the MV, AV, MR orifice and AR orifice (if applicable).

9 $$\text{KE}_{x}= {\int }_{{\rm CS}(t)}\frac{1}{2}\rho |\overrightarrow{v}{\left(t\right)|}^{2}\left(\overrightarrow{v} \cdot \widehat{n}\right)\; dA, x\in \left\{\text{MV,AV,MR,AR}\right\}$$

Lastly, the oscillatory shear index (OSI) was calculated as,^11^

10 $$OSI=\frac{1}{2}\left(1- \frac{\left|{\int }_{0}^{T}\mathop{\text{WSS}}\limits^{\rightharpoonup}dt\right|}{{\int }_{0}^{T}\mathop{\text{WSS}}\limits^{\rightharpoonup}dt}\right)$$

where $\mathop{\text{WSS}}\limits^{\rightharpoonup}$ is the wall shear stress vector.

### Blood Turnover and Washout Efficiency

Fresh blood from mitral inflow was marked by dye through mass-fraction simulations to evaluate blood turnover within the LV chamber. Blood regurgitating from the AV was not marked as it would not introduce fresh blood into the LV. The dye was modelled to have a low diffusivity coefficient of 10^−8^ m^2^/s, which was consistent with the anticipated convection-dominated transport. The simulation was used to gauge oxygen turnover in the LV chamber.

### Statistical Analysis

Anderson–Darling test was performed to check the normality of all continuous variables. Subsequently, paired one-tailed *t*-test was adopted to compare continuous data for pre- and post-FAV if the data had a normal distribution, otherwise, the non-parametric Wilcoxon signed-rank test was performed. *T*-tests for independent samples were performed for comparisons between sub-groups (healthy vs. pre-FAV, healthy vs. post-FAV) if they had normal distribution or the Mann–Whitney *U* test if not. $p<0.05$ was considered statistically significant for all tests.

## Results

### Physiological Features

Patient data regarding complications, outcomes and postnatal procedure details are summarised in Table 1. We did no find any underlying genetic syndrome or extracardiac malformation for any fetus. FAS 2 required treatment for bradycardia during FAV, FAS 1 had LV thrombus that resolved and FAS 3 and 4 had hydrops. Echocardiographic-derived cardiac parameters of the fetuses before and after successful intervention are summarised in Table 2. Pre-FAV LVs were distended, likely due to the high pressure in the LV caused by aortic stenosis. Pre-FAV LVs had long-/short-axis lengths that were substantially greater than the mean of their age cohorts. After FAV, LV dimension significantly reduced, likely due to reduced ventricular afterload from stenosis relief. MV annulus diameter was larger than the mean of their cohort at pre-FAV, but this significantly reduced post-FAV, likely also due to the pressure relief. LV filling times significantly increased with FAV, which was likely due to the decreased LV pressure enabling more inflow.

**Table 2:** Echocardiographic-derived cardiac measurements before and after successful fetal aortic valvuloplasty (FAV) of fetuses with aortic stenosis and evolving hypoplastic left heart syndrome (FAS).

Parameter	Pre-FAV					Post-FAV					*p* value
	FAS 1 (22.57wk)	FAS 2 (24.86wk)	FAS 3 (29.14wk)	FAS 4 (29.00wk)	FAS 5 (30.14wk)	FAS 1 (22.57wk)	FAS 2 (24.86wk)	FAS 3 (29.14wk)	FAS 4 (29.00wk)	FAS 5 (30.14wk)	
LV long axis *Z*-score	1.71	1.08	4.79	2.78	0.49	1.42	0.67	3.35	1.74	− 0.24	0.01
LV short axis *Z*-score	2.48	2.65	5.89	4.06	3.62	2.64	1.12	4.71	3.53	3.07	0.04
RV/LV ratio	1.1	1.06	0.79	0.82	1.05	1.07	1.12	0.84	0.82	1.13	0.10
LV filling time	0.36	0.40	0.41	0.34	0.37	0.41	0.45	0.42	0.37	0.45	0.01
MR V_max_, m/s	3.00	4.20	3.20	3.10	3.51	2.80	3.30	4.00	3.60	3.75	0.39
MV V_max_, m/s	0.35	0.83	1.11	0.80	0.63	0.75	0.87	1.50	0.60	0.55	0.21
AV V_max_, m/s	1.84	3.50	2.44	1.10	1.33	0.75	2.25	2.48	2.10	1.60	0.33
AR V_max_, m/s	–	–	–	–	–	1.87	2.25	2.78	2.50	2.50	–
MV *Z*-score	0.32	0.21	3.26	2.96	3.45	− 0.74	0.21	3.00	2.51	1.98	0.04
AV *Z*-score	− 0.44	0.91	1.68	1.31	1.61	0.77	0.23	1.72	1.36	0.84	0.47

Duration of mitral inflow duration indexed to cardiac cycle length

AV – aortic valve; MV – mitral valve; RV – right ventricle; LV – left ventricle; MR – mitral regurgitation; AR – aortic regurgitation; V_max_ – Maximum velocity

Table 3 compares physiological parameters of the pre-FAV and post-FAV diseased LVs with each other and with healthy fetal LVs. Pre-FAV diseased LVs had abnormally high end-diastolic volumes (EDV), indicating LV dilation, which was likely due to elevated LV pressures caused by aortic stenosis. However, EDV did not always decrease in the post-FAV cohort and remained dilated, suggesting the occurrence of abnormal remodelling caused by the disease. Pre-FAV LVs had abnormally low SV and ejection fraction (EF). FAV improved both measures, suggesting an improvement in LV flow function. LV SV was restored close to healthy hearts.

**Table 3:** Cardiac parameters derived from echocardiography before and after successful fetal aortic valvuloplasty (FAV) of fetuses with aortic stenosis and evolving hypoplastic left heart syndrome (FAS), with healthy comparison

Parameter	Healthy cohort	Disease cohort	
	Overall mean	Mean pre-FAV (*n* = 8)	Mean post-FAV (*n* = 5)
Gestational age, week	26.4 ± 5.1	26.7 ± 3.8	27.1 ± 3.3
Peak AV velocity, m/s	0.74 ± 0.14	1.95 ± 0.81	1.85 ± 0.70^†^
Diastolic E-wave, m/s	0.33 ± 0.14	–	0.63 ± 0.21^†^
Diastolic A-wave, m/s	0.41 ± 0.09	0.84 ± 0.32^†^	0.87 ± 0.42^†^
Peak MR, m/s	–	3.38 ± 0.77	3.54 ± 0.54
Peak AR, m/s	–	–	2.43 ± 0.35
RV SV, ml	1.13 ± 0.57	1.73 ± 1.23	2.02 ± 0.95
LV SV, ml	1.16 ± 0.90	0.76 ± 0.72	1.13 ± 0.64^‡^
RV EDV, ml	2.47 ± 1.29	4.21 ± 2.48	4.16 ± 2.95
LV EDV, ml	2.14 ± 0.16	6.71 ± 5.44^†^	6.64 ± 4.26^†^
LV EF, %	56.2 ± 12.0	12.2 ± 9.9^†^	18.7 ± 11.5^†,‡^
AR volume: LV SV, %	–	–	28.1 ± 15.3
MV annulus diameter, mm	8.32 ± 1.72	10.30 ± 3.38	10.20 ± 3.30
AV annulus diameter, mm	5.12 ± 1.40	4.98 ± 1.06	3.30 ± 0.98
MV flow orifice area, mm^2^*	33.97 ± 21.06	9.44 ± 7.01^†^	12.01 ± 7.41^†^
AV flow orifice area, mm^2^*	20.54 ± 14.39	1.57 ± 2.33^†^	3.11 ± 2.48^†,‡^

AV – aortic valve; MV – mitral valve; RV – right ventricle; SV – stroke volume; LV – left ventricle; EDV – end-diastolic volume; EF – ejection fraction; MR – mitral regurgitation; AR – aortic regurgitation. Disease cohort data from the current study were combined with pre-FAV and healthy cohort data from Wong *et al.*^35^ All data are presented as mean ± standard deviation.

^†^*p* < 0.05 comparing diseased pre-FAV to healthy and post-FAV to healthy; ^‡^*p* < 0.05 comparing diseased pre-FAV and corresponding post-FAV cases.

*Valve orifice areas were backcomputation *via* CFD, by seeking orifice areas that enabled CFD valve velocities to best match Doppler measurements.

Table 3 also shows the valve velocities measured by Doppler, and the valve orifice areas back-computed by simulations to enable a match between simulated valve velocities and Doppler measurements. Pre-FAV AV orifice were drastically smaller than in healthy hearts, reflecting severe stenosis. In post-FAV hearts, AV orifice sizes were roughly doubled from pre-FAV sizes, but it was surprising that the orifice sizes were not much larger and closer to that in healthy hearts. Pre-FAV LVs had AV velocities that were significantly higher than normal LVs due to stenosis. However, post-FAV, there was little change to AV velocities despite reducing the stenosis. This was likely because the post-FAV AV orifice area was still much smaller than normal, and although the widening of the AV orifice would reduce velocities, resolving the stenosis would lead to increased flow rates and thus velocities, negating the reduction. Further, in contrast to pre-FAV cases, AV regurgitation of 2.43 ± 0.35 m/s was observed after FAV in all five cases, suggesting that a technically successful FAV caused damage to the AV.^1^

In terms of MV flow, most pre-FAV LVs had a monophasic inflow pattern, with no distinction between E-wave and A-wave, likely due to poor passive relaxation and diastolic function of the ventricle.^4^ Furthermore, back-computed mitral orifice sizes were substantially smaller than in healthy hearts, while inflow velocities were elevated from that in healthy hearts, suggesting mild stenosis. FAV restored the biphasic inflow waveform in all cases but FAS 1, which is thought to be associated with improved LV diastolic function.^4,34^ However, MV peak velocities remained close to those from pre-FAV and were elevated from healthy LVs, and back-computed MV orifice sizes were only slightly larger compared to pre-FAV state and still substantially smaller than in healthy hearts. This suggested that MV stenosis remained unresolved.

In terms of the RV, pre-FAV RVs were larger than healthy RVs and had higher SV, but no statistical significance was observed due to the high variance, which was partly due to age variability. Remodelling of the RV to compensate for the poor pumping function of the LV caused by aortic stenosis is often observed.^7^ Two days after FAV, there was generally no change to the elevated RV size and function.

### CFD Results: Diastolic Flow Patterns

All CFD simulations were successfully tuned to have a satisfactory match between the simulated peak valve velocities and the clinical Doppler velocity measurements at the valves, with an average percentage error of 3.8 ± 4.2%, as demonstrated in Supplementary Table S1. Comparisons between CFD-simulated peak velocity and Doppler-derived velocity tracings are shown in supplementary Figure S1, showing a satisfactory match, where errors could be due to ultrasound noise affecting motion tracking and imperfection in using the LPM to represent the fetal circulatory system.

**Figure 2:**
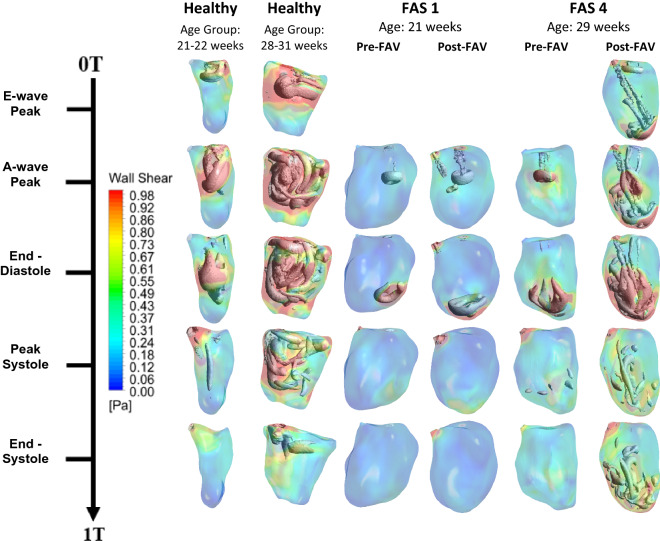
Flow visualisation at different time points of the cardiac cycle using the lambda2 vortex criterion iso-surfaces and wall shear stress color contour plots for two representative healthy LVs and five FAS LVs pre- and post-FAV with data combined from Wong *et al.*^35^ Further results are shown in Supplementary Figure S2 and Supplementary Videos S1–12.

Representative results of LV flow vortex patterns over the cardiac cycle are given in Fig. 2, and details for all cases are given in Supplementary Figure S2 and Supplementary Videos S1–S12. Most subjects in the pre-FAV cohort demonstrated a monophasic diastolic inflow profile. The vortex ring subsequently impinged on the apex causing local WSS elevation around the impingement site. Vorticity dynamics were generally quiescent in pre-FAV LVs due to low flow rates, but there was a wide variability from case to case.

In post-FAV LVs, vorticity dynamics became much more complicated. First, as the intervention led to AR, a vortex ring manifested from the AV and propagated quickly to the apex during early systole. The vortex ring impinged on the apex to cause chaotic secondary vorticity patterns at the apex. In all cases except for FAS 1, there was biphasic mitral inflow, and the peak A-wave velocities were as high as pre-FAV peak velocities. There were thus two MV inflow vortex rings that directly followed the manifestation of the AR vortex ring, with an initial and slower E-wave MV vortex ring preceding a second and faster A-wave vortex ring. These vortex rings moved quickly to the apex as well, even though they were slower than the AR vortex ring. As the MV vortex rings reached the apical region, they impinged on the wall and interacted with the secondary vorticity structures from the earlier AV vortex ring. It increased the complexity and chaos of the vorticity field and enlarged the cloud of secondary vorticity. Compared to the pre-intervention flow field, there were higher vorticity dynamics that covered a larger fluid space, due to the increased flow rates, and because of the additional AV vortex ring. Post-intervention vorticity structures persisted longer and lasted until the end of systole for all cases in contrast to pre-intervention cases. Since the vorticity structures close to the walls were the primary mechanism for inducing WSS, post-interventional cases appeared to have higher overall WSS.

The strong vorticity dynamics manifested despite the viscosity-dominated environment with Reynolds number ranging between 206 and 1360. This suggested the presence of substantial fluid shear stresses and energy losses. It is further noteworthy that AV and MV inflow vortex rings had much instability in the trailing shear layer that led to a pair of secondary rolled-up vortices behind the rings, and this was likely due to the asymmetry of the orifice and the leading vortex ring.^2,20^

### CFD Results: LV Energy Dynamics

**Figure 3:**
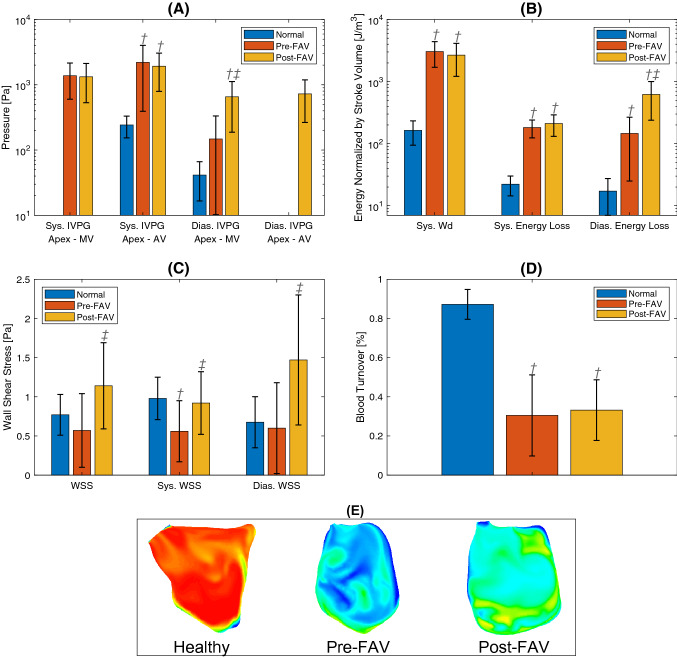
Haemodynamic parameters derived from CFD models. (a) Log plot of systolic (apex-MV/AV) intraventricular pressure gradients (IVPG), and diastolic (apex-MV/AV) IVPG. (b) Log-plot of systolic work done and systolic and diastolic flow energy losses. (c) Time-& surface-averaged wall shear stress (WSS) magnitude over the cardiac cycle, systole and diastole. (d) Percentage of blood turnover measured by the inflow dye mass fraction after three cardiac cycles. (e) End-state in-plane contour maps of the passive dye mass fraction after 3 cardiac cycles for 3 representative cases from each cohort*.* ^†^*p* < 0.05 comparing diseased pre-FAV to healthy and post-FAV to healthy; ^‡^*p* < 0.05 comparing diseased pre-FAV and corresponding post-FAV cases.

Figures 3a and 3b compared the intraventricular pressure gradient (IVPG), systolic work done, systolic and diastolic energy losses between normal, pre-FAV, and post-FAV cohorts. The associated case-specific data are given in Supplementary Table 2.

IVPG was calculated as the peak instantaneous difference in pressure between the apex and the MV or AV orifice, and they were representative of the energy losses associated with in- and outflow. Systolic apex-AV IVPG were elevated in pre-FAV LVs due to aortic stenosis. This did not change after FAV as outflow velocities remained high, due to increased flow rates that occurred with reduced stenosis. Pre-FAV diastolic IVPG was also elevated from that in normal LVs, although no significance was found due to high variability. Post-FAV, diastolic IVPG further increased significantly, due to high-velocity AR that elevated apical pressures, and because post-FAV MV inflow velocities did not decrease from the pre-FAV state. These results suggested that while the pre-FAV flow field had high energy losses, it worsened post-FAV, mainly due to the additional AR and increased LV flow rates.

Similar trends were observed for normalized systolic and diastolic energy loss results. There were significant elevations in pre-FAV LVs compared to normal LVs. After FAV, losses did not improve but became worse. In particular, diastolic energy losses were significantly elevated with FAV. The additional energy losses were associated with additional fluid shear viscous losses consequent to the magnified vorticity dynamics. Similar to IVPG, this was caused by the manifestation of AR coupled with higher flow rates in the LV. Due to these high losses, normalized systolic work done did not change after FAV and remained significantly higher than in normal LVs.

Overall, our results indicated that FAV did not improve the flow energy efficiency but worsened it. Post-FAV energy losses were 37.7 ± 19.8% of systolic work done, compared to 10.8 ± 2.3% for pre-FAV LVs.

### CFD Results: LV Wall Shear Stress

**Figure 4:**
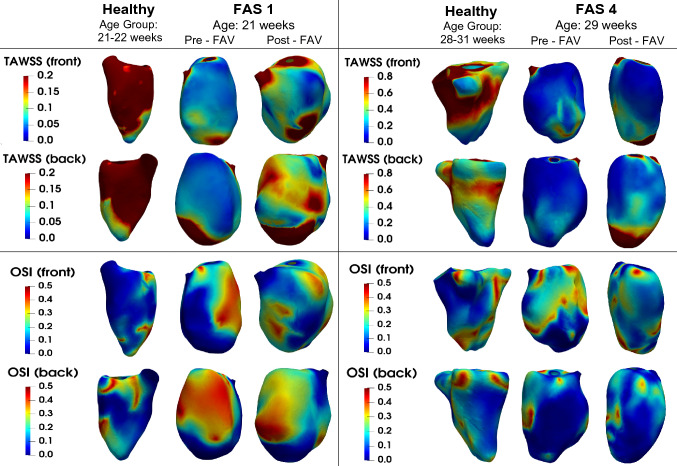
Front and back view of time-averaged wall shear stress (TAWSS) distribution maps and oscillatory shear index (OSI) of representative pre-FAV and post-FAV LVs. Healthy fetal data combined with maps previously published.^35^ Case-specific TAWSS and OSI maps are available in Supplementary Figure S3.

The spatial distributions of time-averaged WSS (TAWSS) and OSI for two age groups (21–22 weeks, 28–31 weeks) are shown for the two representative healthy cases,^35^ and two pre-FAV cases and their corresponding post-FAV cases in Fig. 4. Other cases are shown in Supplementary Figure S3. Regions with high TAWSS generally have a low OSI, and vice versa. In healthy LVs, there were high TAWSS and low OSI in the mid-ventricle zone, due to diastolic vortex rings being wide and slow, and interacting with the mid-ventricular zone more than apical zones. For pre-FAV diseased LVs, high TAWSS and low OSI were generally observed in the apical zone, and this was similarly observed for post-FAV LVs. This spatial pattern was due to fast-moving vortex rings that impinged at the apical site to generate flow disturbances there, which were present in both pre-and post-FAV LVs. Post-FAV cases had higher WSS magnitudes with a larger area of the wall surface having elevated WSS, which was due to the additional and strong AV regurgitation jet and increased MV inflow flow rate, both of which contributed to greater flow disturbances from inflow jet impingement on the apical region.

The overall quantification of time- and surface-averaged WSS is demonstrated in Fig. 3c and Table 4, while case-specific details are given in Supplementary Table 2. We find that in the pre-FAV cases, WSS magnitudes were generally lowered, and the difference was significant for some WSS measures such as systolic time- and surface-averaged WSS and peak diastolic WSS. This significance was observed here but not in our previous study^35^ because we have a larger pre-FAV sample size here. After FAV, WSS were generally and significantly increased for all WSS measures, and the increase was stronger in diastole than systole. Diastolic WSS increased due to the introduction of AR and stronger MV inflow, which caused greater vorticity dynamics and strong shear flow near walls. Systolic WSS also increased because diastolic vortices persisted into systole and because SV and flow rates were higher.

**Table 4:** Computational fluid dynamics-derived haemodynamic parameters before and after successful fetal aortic valvuloplasty (FAV) of fetuses with aortic stenosis and evolving hypoplastic left heart syndrome (FAS), with healthy comparison.

Parameter	Healthy cohort	Disease cohort	
	Overall mean	Mean pre-FAV (*n* = 8)	Mean post-FAV (*n* = 5)
LV systolic (apex – AV) IVPG, Pa	242 ± 89	2208 ± 1815^†^	1925 ± 1131^†^
LV Systolic (apex – MV) IVPG, Pa	–	1377 ± 776	1328 ± 795
LV diastolic (apex – MV) IVPG, Pa	41 ± 25	148 ± 185	655 ± 467^†,‡^
LV diastolic (apex – AV) IVPG, Pa	–	–	727 ± 462
Normalised systolic ejection work done, J/m^3^	164 ± 69	3214 ± 1350^†^	2288 ± 1396^†^
Normalised systolic energy loss, J/m^3^	22 ± 8	182 ± 58^†^	211 ± 80^†^
Normalised diastolic energy loss, J/m^3^	17 ± 10	148 ± 195^†^	655 ± 467^†,‡^
Time- and surface-ave WSS, Pa	0.77 ± 0.26	0.57 ± 0.47	1.14 ± 0.55^‡^
Diastolic peak surface-ave WSS, Pa	0.90 ± 0.38	0.56 ± 0.39^†^	0.92 ± 0.40^‡^
Diastolic time- and surface-ave WSS, Pa	0.67 ± 0.33	0.60 ± 0.58	1.47 ± 0.83^‡^
Systolic peak surface-ave WSS, Pa	1.52 ± 0.36	0.93 ± 0.86	1.88 ± 1.16^‡^
Systolic time- and surface-ave WSS, Pa	0.98 ± 0.27	0.56 ± 0.39^†^	0.92 ± 0.40^‡^

LV – left ventricle; IVPG – intraventricular pressure gradient; WSS – wall shear stress. Disease cohort data from the current study were combined with pre-FAV and healthy cohort data from Wong *et al.*^35^ All data are presented as mean ± standard deviation.

^†^*p* < 0.05 comparing diseased pre-FAV to healthy and post-FAV to healthy; ^‡^*p* < 0.05 comparing diseased pre-FAV and corresponding post-FAV cases.

### CFD Results: Blood Turnover and Washout Efficiency

Figure 3d compares the percentage of fresh blood introduced into the LV in normal, pre-FAV and post-FAV cases after three cardiac cycles. Figure 3e showed that the poor blood turnover efficiency demonstrated by the pre-FAV cases did not significantly improve after FAV. This was because MV inflow only improved marginally, and most of the improvement in SV after FAV was accounted for by AR, which would not introduce fresh blood to the LV. This had potential implications for oxygen content in the LV chamber and occurrences of endocardial fibroelastosis, as our discussions below will explain.

## Discussion

Our image-based flow simulations enabled the characterization of the acute changes to LV flow patterns, WSS and energy dynamics caused by FAV. Results showed a restoration of SV to the normal level and improvements to the EF. Even though much of the additional SV would be due to AR, these improvements were likely to restore some myocardial deformational stimuli, increase flow stimuli to heart valves and the aorta, and cause a transition from the abnormal retrograde systolic transverse aortic arch flow to the normal antegrade one.^25^ Our results also showed increased WSS stimuli to the LV walls. These positive changes are likely to improve morphological development.

Surprisingly, we found that FAV worsened the already poor flow energy efficiency in the LV and caused additional and very excessive energy losses in LV flow, which could be up to 37.7% of the work done by the heart on LV fluid, and which was likely the reason that systolic work done did not normalize directly after FAV. We found that the primary reason for this was the introduction of AR, which was very severe and had high velocities, coupled with the increased amount of MV inflow. This generated a highly chaotic diastolic flow pattern that persisted throughout the cardiac cycle, leading to excessive viscous energy losses.

In a FAV intervention, one important consideration is that the stenosis should be sufficiently resolved, as there are cases of AV re-stenosis that required a second intervention,^5,29^ which is highly undesirable as the interventions pose significant risks to the fetuses. Consequently, balloon sizes are typically chosen to be large,^1,15^ which would inevitably cause severe AR in most cases. The resulting diastolic dysfunction and high energy burden on the heart might be detrimental to the heart. Regarding this issue, fetal transcatheter aortic valve replacement (TAVR), might mitigate this. There have been recent advances in developing fetal TAVR, Zakko *et al.* demonstrated the transcatheter delivery of a tissue-engineered heart valve in a fetal ovine model, which survived to birth.^37^

On the other hand, there can be opposing considerations, and it could be that there was no need to address the AR. Firstly, several weeks after FAV, a reduction of AR was frequently observed, perhaps due to AV remodelling, and the poor diastolic function and excessive energy losses would then be resolved. Secondly, AR enhanced the SV of the heart and allowed the LV myocardium to undergo greater deformation, and this could be an important biomechanical stimuli to stimulate continued LV growth and the normalization of LV morphological development. Past studies have found that increase in LV strains after FAV correlated with a BV outcome as opposed to a UV outcome,^8^ corroborating this notion. However, there is insufficient data to prove this, and further studies are needed to verify this effect.

Our results also indicated that FAV did not resolve the stenosis as extensively as was hoped. Our computations showed that although the AV flow orifice was doubled on average after the intervention, it is still about five times smaller than that in the normal heart. This was surprising considering the balloon was chosen to be close to the annulus size and could be because the post-FAV AV had an abnormal structure that would still pose an obstruction to flow. Again, further studies are also required here to understand the reason for poorer than expected performance, and to devise strategies to increase post-FAV orifice sizes.

In terms of WSS, we find that the FAV significantly increased WSS, and restored it to levels exceeding those in normal LV. We hypothesize that this would induce improved LV growth to lead to a biventricular birth outcome, as WSS is thought to be a stimulus for gestational cardiac growth. The additional WSS that were imposed on heart valves and the aorta may also improve their growth rate to reach biventricular outcomes.^10,16^

However, our simulations showed that FAV did not significantly improve LV blood turnover in the first 2 days after the procedure. This suggested that the oxygen content in the LV chamber would not have improved drastically, which may have implications for endocardial fibroelastosis (EFE). EFE is prevalent in FAS, appearing as a hyperechogenic endocardial lining.^4,18^ Recent work showed the role of aberrant endothelial-to-mesenchymal transition (EndMT) in EFE formation,^36^ and that hypoxia-induced by left atrial ligation models of chick embryos could cause myocardial fibrosis.^23^ According to this theory, our results would suggest that FAV will not decrease the risks of EFE in FAS LVs.

Our current study has several important limitations. Firstly, the study is limited to the acute changes in flow biomechanics consequent to FAV. In many fetal cases, positive AV remodelling occurs several weeks after FAV, but this was not analysed here. Secondly, the fetal heart has much plasticity and has the potential for a wide range of remodelling for the remainder of gestation, and this was not studied. As such, an important future work would be to understand the growth and remodelling of the heart and to investigate if biomechanics play a role in influencing it. It would further be important to determine factors that are associated with UV or BV birth outcomes but our study did not address this. Thirdly, our results suggested that high energy losses during diastole were related to AR, which from our clinical experience, occurs in 84% of FAV cases. However, the absence of AR is also possible after FAV, and such cases are not analyzed here. We speculate that in such cases, energy losses would remain similar to pre-FAV levels, as transaortic velocities tend to remain the same across FAV, but future work is necessary to ascertain this. Another limitation was our idealization of the MV to be an abruptly opened or closed orifice without valve leaflets. This was due to an inability of fetal echo imaging to capture the valve structure and dynamics and thus a lack of information on what caused the mild mitral stenosis. However, we believe that this idealization would not affect results concerning the fast-moving diastolic vortex rings, the associated WSS patterns and magnitude and energy losses. Finally, image noise and signal losses could also lead to errors in segmentation and motion tracking.

In conclusion, we examined the effect of FAV on LV flow dynamics and energy dynamics through patient-specific flow simulations. We found that FAV improved the SV and EF of the LV, and enhanced the WSS stimuli to LV walls, but it caused AR, which led to a chaotic diastolic flow pattern, highly unfavourable flow energy efficiency and very excessive energy losses directly after FAV. Further, we estimated that the AV orifice was only marginally improved by FAV, and the poor blood turnover of FAS LVs was not significantly improved.

## Supplementary Information

Below is the link to the electronic supplementary material.

Supplementary file1 (MP4 3095 kb)

Supplementary file2 (MP4 3215 kb)

Supplementary file3 (MP4 3094 kb)

Supplementary file4 (MP4 3041 kb)

Supplementary file5 (MP4 3206 kb)

Supplementary file6 (MP4 3181 kb)

Supplementary file7 (MP4 2529 kb)

Supplementary file8 (MP4 3107 kb)

Supplementary file9 (MP4 2680 kb)

Supplementary file10 (MP4 2628 kb)

Supplementary file11 (DOCX 9243 kb)
