# Supplementary material for: Fluid Mechanical Effects of Fetal Aortic Valvuloplasty for Cases of Critical Aortic Stenosis with Evolving Hypoplastic Left Heart Syndrome
Source: Ann Biomed Eng. 2023 Feb 13;51(7):1485–98. doi: 10.1007/s10439-023-03152-x (PMC10264284; doi:10.1007/s10439-023-03152-x)
Supplement: Supplementary file 11 — Supplementary file11 (DOCX 9243 kb) [file 10439_2023_3152_MOESM11_ESM.docx]

**Supplementary Text for Manuscript:**

**Fluid Mechanical Effects of Fetal Aortic Valvuloplasty for Cases of Aortic Stenosis and Evolving Hypoplastic Left Heart Syndrome.**

Hong Shen Wong^1^*, Binghuan Li^1^*, Andreas Tulzer^2^, Gerald Tulzer^2^, Choon Hwai Yap^1^

1 Department of Bioengineering, Imperial College London, Exhibition Road, London, SW7 2AZ, United Kingdom

2 Children’s Heart Center Linz, Department of Pediatric Cardiology, Kepler University Hospital, Medical Faculty of the Johannes Kepler University, Krankenhausstrasse 26-30, Linz 4020, Austria

* Authors have equal contributions

***Supplementary Table S1:*** *Case-specific comparison between computational fluid dynamics simulation result and clinical measurements of valve velocities.*

| ***Parameter*** | ***Pre-FAV Cohort*** | | | | | | | | | | ***Post-FAV Cohort*** | | | | | | | | | |
| --- | --- | --- | --- | --- | --- | --- | --- | --- | --- | --- | --- | --- | --- | --- | --- | --- | --- | --- | --- | --- |
|  | FAS 1  (22.57 wk) | | FAS 2  (24.86 wk) | | FAS 3  (29.14 wk) | | FAS 4  (29 wk) | | FAS 5  (30.14 wk) | | FAS 1  (22.57 wk) | | FAS 2  (24.86 wk) | | FAS 3  (29.14 wk) | | FAS 4  (29 wk) | | FAS 5  (30.14 wk) | |
|  | CFD | Dop. | CFD | Dop. | CFD | Dop. | CFD | Dop. | CFD | Dop. | CFD | Dop. | CFD | Dop. | CFD | Dop. | CFD | Dop. | CFD | Dop. |
| Peak AV, m/s | 1.80 | 1.84 | 3.44 | 3.50 | 2.38 | 2.44 | 1.16 | 1.10 | 1.27 | 1.33 | 0.79 | 0.75 | 2.23 | 2.25 | 2.59 | 2.48 | 2.06 | 2.10 | 1.58 | 1.60 |
| Diastolic E, m/s |  |  |  |  |  |  |  |  |  |  |  |  | 0.66 | 0.60 | 0.77 | 0.80 | 0.52 | 0.50 | 0.60 | 0.51 |
| Diastolic A, m/s | 0.38 | 0.35 | 0.87 | 0.83 | 1.05 | 1.11 | 0.94 | 0.80 | 0.60 | 0.63 | 0.70 | 0.55 | 0.86 | 0.87 | 1.60 | 1.50 | 0.60 | 0.60 | 0.60 | 0.55 |
| MV Regurgitation, m/s | 3.04 | 3.00 | 4.22 | 4.20 | 3.15 | 3.20 | 3.12 | 3.10 | 3.77 | 3.51 | 2.80 | 2.80 | 3.35 | 3.30 | 4.29 | 4.00 | 3.62 | 3.60 | 3.64 | 3.75 |
| AV Regurgitation, m/s |  |  |  |  |  |  |  |  |  |  | 1.92 | 1.87 | 2.26 | 2.25 | 2.83 | 2.78 | 2.55 | 2.50 | 2.61 | 2.50 |

*AV - aortic valve; CFD – Computational Fluid Dynamics; Dop. – Doppler Ultrasound measurements.*

***Supplementary Table S2:*** *Case-specific comparison of computational fluid dynamics simulated cardiac parameters and clinical measurements of valve velocities before and after fetal aortic valvuloplasty (FAV).*

| **Parameter** | **FAS 1**  22.57 week | | **FAS 2**  24.86 week | | **FAS 3**  29.14 week | | **FAS 4**  29 week | | **FAS 5**  30.14 week | | **P-Value** |
| --- | --- | --- | --- | --- | --- | --- | --- | --- | --- | --- | --- |
|  | ***Pre – FAV*** | ***Post - FAV*** | ***Pre - FAV*** | ***Post - FAV*** | ***Pre - FAV*** | ***Post - FAV*** | ***Pre - FAV*** | ***Post - FAV*** | ***Pre - FAV*** | ***Post - FAV*** |  |
| Peak AV velocity, m/s | 1.79 | 0.79 | 3.15 | 2.23 | 2.38 | 2.12 | 1.16 | 2.06 | 1.27 | 1.58 | 0.358 |
| Diastolic E – wave, m/s | - | - | - | 0.60 | - | 0.90 | - | 0.40 | - | 0.60 | - |
| Diastolic A – wave, m/s | 0.38 | 0.70 | 0.87 | 0.86 | 1.05 | 1.34 | 0.94 | 0.60 | 0.64 | 0.60 | 0.287 |
| Peak MV regurgitation, m/s | 3.04 | 2.80 | 4.22 | 3.35 | 3.15 | 3.84 | 3.12 | 3.62 | 3.77 | 3.64 | 0.413 |
| Peak AV regurgitation, m/s | - | 1.92 | - | 2.26 | - | 2.92 | - | 2.55 | - | 2.61 | - |
| RV stroke volume, ml | 1.09 | 0.87 | 0.82 | 1.52 | 1.56 | 3.39 | 2.30 | 2.43 | 2.34 | 1.91 | 0.19 |
| LV stroke volume, ml | 0.14 | 0.16 | 0.64 | 0.94 | 1.20 | 1.86 | 0.68 | 1.22 | 0.84 | 1.48 | **0.011** |
| RV EDV, ml | 2.91 | 3.05 | 1.84 | 3.30 | 3.16 | 6.98 | 5.29 | 5.23 | 6.13 | 7.25 | 0.200 |
| LV EDV, ml | 2.99 | 2.53 | 2.23 | 2.53 | 17.40 | 12.61 | 11.1 | 8.23 | 6.68 | 7.32 | 0.120 |
| LV Ejection Fraction, % | 4.63 | 6.37 | 28.7 | 37.3 | 6.90 | 14.76 | 6.15 | 14.84 | 12.6 | 20.3 | **<0.01** |
| AV regurgitation volume: LV SV, % | - | 10.2 | - | 16.5 | - | 29.3 | - | 35.4 | - | 48.9 | **-** |
| MV annulus, mm | 68 | 57 | 75 | 76 | 135 | 132 | 129 | 123 | 144 | 120 | 0.059 |
| AV annulus, mm | 32 | 38 | 43 | 40 | 58 | 59 | 55 | 56 | 60 | 55 | 0.500 |
| MV flow orifice area, mm^2^ | 3.50 | 2.46 | 7.40 | 11.31 | 16.46 | 9.82 | 9.82 | 13.46 | 20.41 | 23.01 | 0.404 |
| AV flow orifice area, mm^2^ | 0.11 | 1.82 | 0.18 | 0.59 | 0.55 | 3.97 | 1.71 | 2.19 | 6.58 | 6.99 | **0.047** |
| LV systolic (Apex – AV) IVPG, Pa | 1607 | 331 | 5884 | 2601 | 2930 | 3227 | 697 | 2154 | 831 | 1313 | 0.301 |
| LV systolic (Apex – MV) IVPG, Pa | 949 | 197 | 2480 | 1740 | 1340 | 2310 | 838 | 1400 | 345 | 995 | 0.363 |
| LV diastolic (Apex – AV) IVPG, Pa | - | 228 | - | 960 | - | 447 | - | 601 | - | 1401 | - |
| LV diastolic (Apex – MV) IVPG, Pa | 22 | 220 | 194 | 899 | 98 | 278 | 105 | 539 | 47 | 1340 | **0.026** |
| Normalised Systolic Ejection Work Done, J/m^3^ | 2846 | 461 | 4178 | 3732 | 2827 | 3632 | 2534 | 3655 | 1800 | 1885 | 0.334 |
| Normalised Systolic Energy Loss, J/m^3^ | 163 | 74 | 263 | 239 | 216 | 276 | 161 | 253 | 124 | 212 | 0.237 |
| Normalised Diastolic Energy Loss, J/m^3^ | 34 | 156 | 135 | 284 | 171 | 917 | 97 | 733 | 71 | 1009 | **0.018** |
| Haemodynamic Inefficiency, % | 6.9 | 49.9 | 8.32 | 14 | 13.7 | 32.8 | 10.2 | 27 | 10.8 | 64.8 | **0.039** |
| Time- & Surface-Ave. WSS (Pa) | 0.15 | 0.27 | 1.02 | 1.38 | 0.56 | 1.35 | 0.39 | 1.01 | 0.52 | 1.72 | **0.014** |
| Diastolic Peak Surface-Ave. WSS (Pa) | 0.18 | 0.48 | 2.17 | 3.05 | 0.91 | 2.40 | 0.56 | 1.53 | 1.00 | 3.68 | **0.017** |
| Diastolic Time- & Surface-Ave. WSS (Pa) | 0.14 | 0.28 | 1.21 | 1.84 | 0.46 | 1.47 | 0.38 | 1.22 | 0.38 | 2.54 | **0.023** |
| Systolic Peak Surface-Ave. WSS (Pa) | 0.23 | 0.33 | 1.37 | 2.41 | 1.03 | 1.88 | 0.50 | 1.35 | 1.00 | 3.44 | **0.025** |
| Systolic Time- & Surface-Ave. WSS (Pa) | 0.17 | 0.27 | 0.84 | 1.02 | 0.62 | 1.25 | 0.40 | 0.83 | 0.76 | 1.24 | **0.011** |

*P-value<0.05, paired comparison between diseased pre-FAV and post-FAV.
AV - aortic valve; MV – mitral valve; RV - right ventricle; LV - left ventricle; EDV - end-diastolic volume; IVPG – Intraventricular pressure gradients; WSS – Wall Shear Stress; Surface-Ave. – Surface-averaged.*

|  | ***Supplementary Figure S1:*** *CFD-obtained valve maximum velocities compared with Doppler-obtained velocities for the (i) Aortic antegrade outflow, (ii) Mitral inflow and (iii) Mitral regurgitation and, additionally (iv) Aortic regurgitation if present, for a (A) pre-FAV case, FAS 3 and a (B) post-FAV case, FAS 4.* |
| --- | --- |

***Supplementary Figure S2:*** *Wall shear stress color contour and λ_2_ iso-velocity surfaces for pre-& post-FAV cases*

***Supplementary Figure S3:*** *Case-specific surface* *colour contours* *maps of time-averaged WSS (TAWSS) and oscillatory shear index (OSI) distribution before and after fetal aortic valvuloplasty (FAV)*
